# Retro-mode imaging and fundus autofluorescence with scanning laser ophthalmoscope of retinal dystrophies

**DOI:** 10.1186/1471-2415-12-8

**Published:** 2012-05-15

**Authors:** Battaglia Parodi Maurizio, Iacono Pierluigi, Kontadakis Stelios, Vergallo Stefano, Cascavilla Marialucia, Zucchiatti Ilaria, Bandello Francesco

**Affiliations:** 1 Department of Ophthalmology, Vita-Salute University, San Raffaele Scientific Institute, Milan, Italy; 2 G. B. Bietti Foundation, IRCCS (Istituto di Ricovero e Cura a Carattere Scientifico), Rome, Italy; 3 Clinica Oculistica, Santa Maria della Misericordia Hospital, Udine, Italy

## Abstract

**Background:**

Retinal dystrophies display a considerably wide range of phenotypic variability, which can make diagnosis and clinical staging difficult. The aim of the study is to analyze the contribution of retro-mode imaging (RMI) and fundus autofluorescence (FAF) to the characterization of retinal dystrophies.

**Methods:**

Eighteen consecutive patients affected by retinal dystrophies underwent a complete ophthalmological examination, including best corrected visual acuity with ETDRS charts, blue-light fundus autofluorescence, (BL-FAF), near-infrared fundus autofluorescence (NIR-FAF), and RMI. The primary outcome was the identification of abnormal patterns on RMI. The secondary outcome was the correlation with the findings on BL-FAF and NIR-FAF.

**Results:**

Overall, the main feature of RMI is represented by a pseudo-3D pattern of all the lesions at the posterior pole. More specifically, any accumulation of material within the retina appears as an area of elevation of different shape and size, displaying irregular and darker borders. No precise correlations between RMI, BL-AF, and NIR-AF imaging was found.

**Conclusions:**

RMI and FAF appear to be useful tools for characterizing retinal dystrophies. Non-invasive diagnostic tools may yield additional information on the clinical setting and the monitoring of the patients.

## Background

A peculiar trait of retinal dystrophies is their considerable range of phenotypic variability, which sometimes makes diagnosis and clinical staging difficult. Many attempts have been made over the past decade to characterize each dystrophy by more precise diagnostic techniques, including fundus autofluorescence (FAF) and optical coherence tomography (OCT) [1-3]. As a result, the study of clinical features and morpho-functional correlations of the retinal dystrophies has greatly improved. Nevertheless, a complete knowledge of all the clinical manifestations is yet to be achieved. Retro-mode imaging (RMI) has recently been shown to offer the best method of outlining the distinctive clinical features of several chorio-retinal disorders [4-8]. RMI is performed by a scanning laser ophthalmoscope (SLO) working at infrared wavelength to produce a pseudo-3D image. The aim of the present study is to describe patterns typical of retinal dystrophies on RMI, and to compare them with the findings obtained on FAF.

## Methods

All the consecutive patients affected by retinal dystrophies referred to our department of Ophthalmology for a clinical evaluation from May 2010 to September 2010 were prospectively considered for the study. This research was approved by the institutional review board and adhered to the tenets of the Declaration of Helsinki. Each patient provided signed informed consent to the study.

The inclusion criterion was the diagnosis of retinal dystrophy. Exclusion criteria were the presence of media opacities and the identification of any other ocular or systemic disease able to give rise to chorioretinal alterations.

Each patient underwent a complete ophthalmic examination, including measurement of visual acuity, FAF and RMI. Best-corrected visual acuity (BCVA) was determined with standard Early Treatment Diabetic Retinopathy Study charts as the total number of correct letters identified at 4 m plus 30.

RMI was obtained using a F10 SLO (Nidek, Padova, Italy) [7-9]. In brief, the F10 can obtain images of the deeper retinal layers employing a 790 nm infrared laser; it is equipped with a retro-mode aperture consisting of a central stop associated with a laterally oriented oval-shaped opening, which prevents the direct light reflection crossing and allows the scattered light from only one direction to pass through the lateral aperture. The detector acquires images from surface to deep retinal layers through the lateral aperture and provides a final image appearing as a shadow of the silhouetted retino-choroidal details. This imaging technique produces pseudo-3D features offering a new means of detecting abnormalities in the deeper retinal layers and choroid.

Blue-light fundus autofluorescence (BL-FAF) was recorded using a confocal scanning laser ophthalmoscope (Heidelberg Retina Angiograph, HRA, Heidelberg Engineering, Heidelberg, Germany) using a 30° field of view and 512 × 512-pixel resolution. The optical and technical performance of the confocal scanning laser ophthalmoscope have been previously reported [9]. In brief, the instrument uses blue laser light at 488 nm for excitation and a barrier filter at 500 nm to limit the captured light to autofluorescent structures. Near-infrared fundus autofluorescence (NIR-FAF) was obtained using the standard HRA angiograph system, using an excitation power and wavelength of 789 nm and a detection filter of >800 nm normally employed in indocyanine green angiography [3,10,11]. Five to nine images were taken and averaged to obtain a single image for BL-AF and NIR-AF.

The primary outcome was the identification of abnormal patterns on RMI. The secondary outcome was the correlation with the findings on BL-FAF and NIR-FAF.

The study adhered to the tenets of the Declaration of Helsinki for research involving human subjects and was approved by Ethics Committee of the Vita-Salute University, San Raffaele Scientific Institute.

## Results

A total of 18 patients with retinal dystrophies participated in the study. We included 2 patients with Best Vitelliform Macular Dystrophy (BVMD), 5 patients with Autosomal Recessive Stargardt Disease-Fundus Flavimaculatus (STGD1), 8 patients with Pattern Dystrophy (PD) of the Retinal Pigment Epithelium, 2 patients with Choroideremia (CHM), and 1 patient with Benign Concentric Annular Macular Dystrophy (BCAMD). All the diagnoses of the retinal dystrophies were confirmed by molecular genetic analysis.

### Autosomal recessive Stargardt disease-fundus flavimaculatus

The 5 patients affected by STGD1 included 4 females and 1 male, with ages ranging from 22 to 67 years, and BCVA from 20/50 to 20/400. The diagnosis of STGD1 was confirmed by the identification of 2 compound heterozygous mutations in the ABCA4 gene.

**Figure 1:**
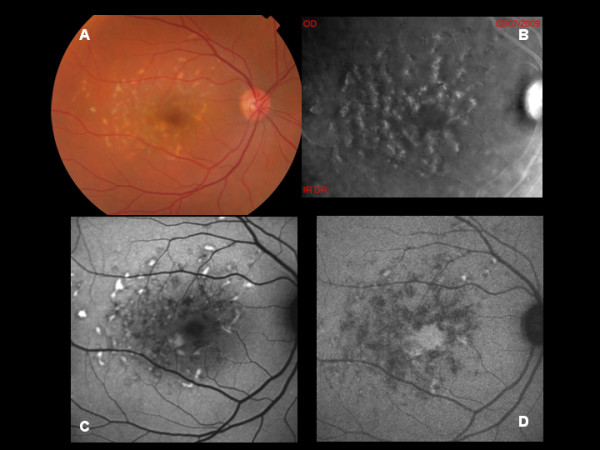
**A: Color photograph of Stargardt disease with many flecks at the posterior pole. B:** Multiple small elevated lesions are detectable on retromode imaging and most of the lesions correspond to the flecks visible at biomicroscopy even though not all. **C:** BL-AF shows areas of focal increased signal in most of the flecks, whereas NIR-AF (**D**) discloses mostly hypo-autofluorescent lesions.

**Figure 2:**
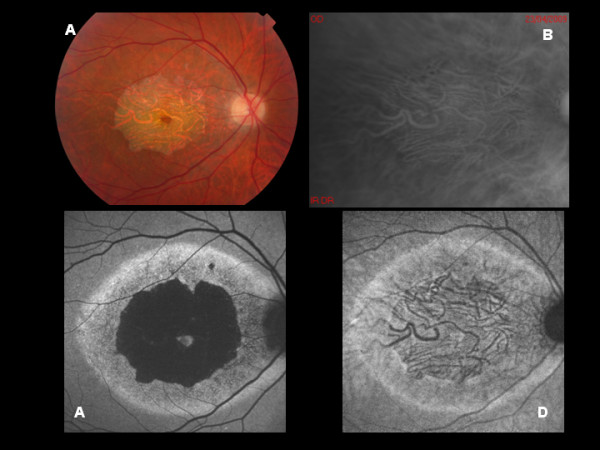
**A: Color photograph of Stargardt disease at an advanced stage. B:** Retromode imaging shows a well-defined visualization of the choroidal vasculature corresponding to the atrophy on biomicroscopy. **C:** BL-AF reveals a central decreased signal surrounded by a hyper-auotofluorescent ring similar to that detectable on NIR-AF (**D**).

All patients displayed specific abnormalities on RMI; especially, multiple, small, elevated lesions. Most of the lesions visualized on RMI corresponded to the flecks identified on biomicroscopy, but some smaller lesions were visible just on RMI (Figure 1). The parallel with FAF disclosed interesting features. In particular, BL-FAF revealed areas of focal increased signal in most of the flecks, but it also showed some hypo-autofluorescent flecks. On the other hand, NIR-FAF disclosed mostly hypo-autofluorescent lesions corresponding to the biomicroscopically visible flecks, along with some lesions displaying a focally increased response. In one case characterized by advanced STGD1 with a macular atrophy, RMI showed a clear demarcation of the choroidal vasculature (Figure 2). BL-FAF revealed a central decreased signal surrounded by a hyper-auotofluorescent ring, which turned out to be similar to the image on NIR-FAF. Interestingly, there was no evidence of elevated lesions corresponding to flecks, probably due to the atrophic changes.

### Best vitelliform macular dystrophy

**Figure 3:**
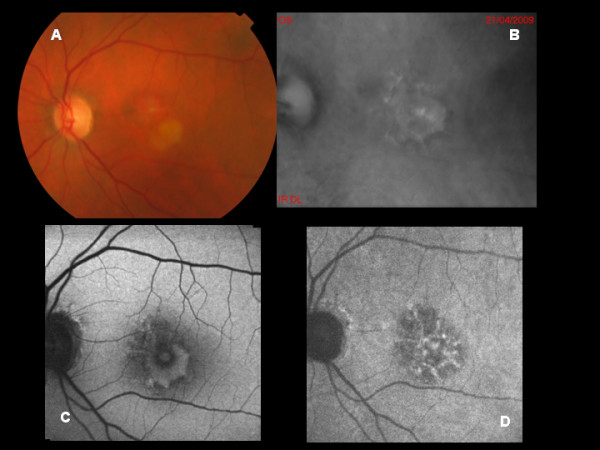
**A: Color photograph of a case of Best vitelliform macular dystrophy. B:** Retromode imaging discloses the presence of a polygonal elevated area in the macula corresponding to the vitelliform material deposition on ophthalmoscopy. **C:** BL-AF shows an increased FAF especially in the inferior part of the lesion, together with some hypo-autofluorescent portions. **D:** NIR-AF shows a wide hypo-autofluorescent halo centered by a hyper-autofluorescent net.

The 2 cases affected by BVMD included a 20-year-old male (BCVA of 20/60 in both eyes), and a 28-year-old female (BCVA of 20/125 in both eyes). Genetic analyses confirmed the mutation in the bestrophin gene. RMI disclosed the presence of a polygonal elevated area in the macular area, corresponding to the vitelliform material deposition found on ophthalmoscopy, which appeared brighter in the centre and darker at the margins (Figure 3). This lesion on the whole looked greater on RMI than on biomicroscopy and showed uneven extensions at its boundaries. BL-FAF showed an overall increased FAF, associated with some hypo-autofluorescent portions. NIR-FAF disclosed a wide hypo-autofluorescent halo centered by a hyper-autofluorescent net. Comparison of the images confirmed that RMI revealed a larger lesion than BL-FAF and NIR-FAF.

### Pattern dystrophy of the retinal pigment epithelium

**Figure 4:**
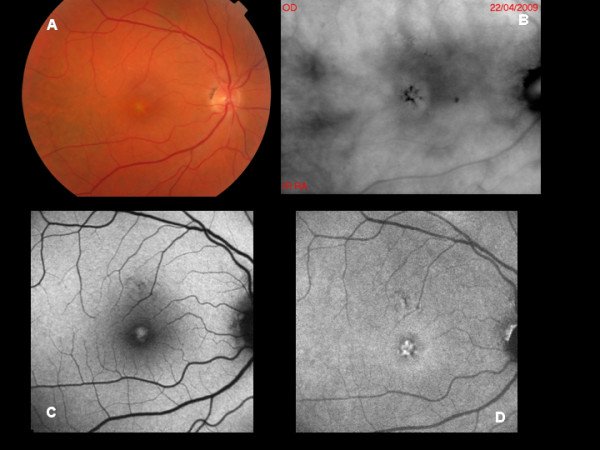
**A: Color photograph of Adult-onset foveomacular vitelliform dystrophy. B:** Retromode imaging shows focally risen lesions with darker borders. **C:** BL-AF reveals focally increased signal corresponding to the accumulation of abnormal material. **D:** NIR-AF displays a more intensely hyper-autofluorescent lesion.

The 8 patients affected by PD included 6 cases of Adult-Onset Foveomacular Vitelliform Dystrophy and 2 cases of Reticular Dystrophy. More specifically, we included 6 males and 2 females, with age ranging from 35 to 55 years, and BCVA from 20/60 to 20/30. Genetic analyses identified mutations in the RDS gene. In all the cases RMI revealed focally risen lesions with darker borders (Figure 4). A single case displayed small elevated dots (corresponding to biomicroscopically visible drusen), together with a single dark spot (with no equivalent lesion on biomicroscopy and FAF). BL-FAF revealed focally increased signal corresponding to the accumulation of abnormal material, whereas NIR-FAF displayed a more intensely hyper-autofluorescent lesion. It is noteworthy that the lesions identified on RMI turned out to be larger than those visualized on both biomicroscopy and on the two FAF techniques.

### Choroideremia

**Figure 5:**
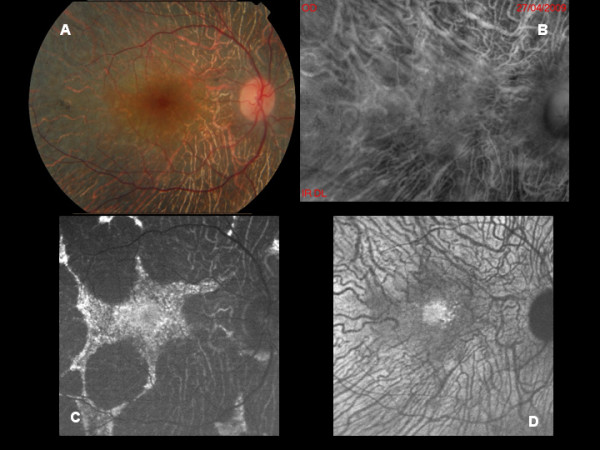
**A: Color photograph of a patient affected by Choroideremia.B:** Retromode imaging is able to delineate the choroidal vessels. **C:** BL-AF shows an irregular area with hyper-autofluorescence with non-homogeneous appearance, possibly indicating the RPE impairment. **D:** NIR-AF discloses a weak hypo-autofluorescence corresponding to the spared retina, associated with a round region with more intense signal.

The 2 patients affected by Choroideremia were a 27- and a 31-year old male, with a bilateral BCVA of 20/100 and 20/80, respectively. The diagnosis was confirmed by identifying mutations in the CHM gene. RMI showed an extensive visualization of the choroidal vessels (Figure 5). The central retina, which was spared by the degenerative process, looked like a thin veil and permitted the underlying vasculature to be partially identified. BL-FAF showed an irregular area with hyper-autofluorescence with uneven appearance, possibly indicating the RPE impairment. NIR-FAF disclosed a weak hypo-autofluorescence corresponding to the spared retina, associated with a round region with more intense signal.

### Benign concentric annular macular dystrophy

The single 57-year-old patient affected by BCAMD had a BCVA of 20/400 in both eyes. Linkage analysis showed linkage on chromosome 6p12.3-q16. RMI revealed a precise visualization of the choroidal vessels, the central area being spared. BL-AF showed an annular hypo-autofluorescence with relatively spared central retina. NIR-AF disclosed a hyper-autofluorescence corresponding to the spared retina.

## Discussion

Macular dystrophies are a heterogeneous group of hereditary disorders involving the macular area at different levels and with variable severity. Diagnostic tools, including fluorescein angiography, FAF, electrofunctional tests, and OCT can provide information regarding the morphological changes characterizing each dystrophy. Infrared examination of the human ocular fundus has provided interesting results in the past [12,13]. More recently, the F10 SLO has been used non-invasively to examine the posterior pole with a retro-mode technique at an infrared wavelength, providing accurate characterization of the retinochoroidal structures [4-8]. The F10 is based on a newly developed RMI procedure using an infrared laser to provide a pseudo three-dimensional image.

The pseudo-3D image is obtained by assembling multiple pictures generated by the retinal layers at different depths and collected by the F10-detector through the retro-mode aperture. This consists of a central stop blocking the direct light reflection and a laterally oriented oval-shaped opening, which allows the scattered light spreading in only one direction to cross.

In previous reports, RMI was able to characterize the cystoid macular edemas secondary to polypoidal choroidal vasculopathy, retinitis pigmentosa or retinal vascular disorders, including diabetic maculopathy and retinal vein occlusion [4,5]. Tanaka et al have described a specific ‘fingerprint’ pattern related to macular retinoschisis in myopic eyes, which consists of radiating retinal striae centered on the fovea and many light dots and lines that run in parallel to the striae or form a whorled pattern surrounding the radiating striae [6]. In eyes affected by dry age-related macular degeneration, RMI featured the progressive enlargement and confluence of drusen over a short term observation, suggesting a possible role in the monitoring of subtle drusen changes in the progression of AMD [8].

Our results reveal that RMI is also able to detect abnormalities in retinal dystrophies. In particular, the main finding of RMI is a pseudo-3D pattern of all the lesions at the posterior pole. Any accumulation of material within the retina appeared as an elevated area, with different shapes and sizes, showing irregular and darker borders. On the other hand, atrophic regions turned out to be accurately outlined by the precise visualization of the choroidal vasculature, both in the macula and outside the macular region when the fovea was spared.

Comparison with other imaging techniques can be interesting. Indeed, BL-FAF is considered to be related to the amount of lipofuscin within the RPE cells [9], whereas NIR-AF patterns have been interpreted as corresponding to the extent of melanin deposition [3,10,11]. Interestingly, we did not find a clear correlation between RMI, and BL-FAF or NIR-FAF imaging. Bearing in mind that both BL-FAF and NIR-FAF signals correspond to the fluorescence of specific molecules (lipofuscin and melanin, respectively), the presence of other molecules contributing to the whole mass of each specific alteration cannot be visualized by the two FAF techniques. RMI thus seems to be a technique able to depict the full extension of the abnormal material amassed. Moreover, RMI provides a more precise imaging of the elevated lesions than biomicroscopic examination, precisely detecting even small alterations that can be missed in a simple ophthalmoscopic examination.

OCT can also produce 3D imaging of retinal abnormalities, providing insights useful in the diagnosis of macular diseases. In view of the fact that some alterations are located at a different depth of the retina, certain authors have argued that it is not always easy to see all the changes in a single OCT section [5,14]. OCT and RMI may thus be considered complementary techniques, together providing a fast and non-invasive examination of the fundus.

## Conclusions

RMI appears to be a useful tool for the characterization of retinal dystrophies. RMI may yield additional information in the diagnostic setting of macular lesions and may represent a practical diagnostic tool for cross-sectional or family studies and for the progressive monitoring of retinal dystrophy.

## Competing interest

The authors have no proprietary/ financial interest in any of the products mentioned in the study. No financial support was received.

## Authors’ contributions

MBP, PI, KS, MLC and ZI contributed to the concept and design, writing the article, data analysis and interpretation, data collection. FB contributed to the critical revision and final approval of the article. All authors read and approved the final manuscript.

## Pre-publication history

The pre-publication history for this paper can be accessed here:

http://www.biomedcentral.com/1471-2415/12/8/prepub
